# Expanding the Clinical Spectrum of Poikiloderma with Neutropenia: A Case Report of Progressive Tendon Contractures

**DOI:** 10.51894/001c.164422

**Published:** 2026-08-01

**Authors:** Yuri Alonte, Ibrahim Saad, Sudershan Grover

**Affiliations:** 1 Henry Ford Warren Hospital

## 58

### Background

Poikiloderma with Neutropenia (PN), also known as Navajo poikiloderma, is a rare autosomal recessive genodermatosis characterized by early-onset poikiloderma, chronic neutropenia, recurrent infections, and additional ectodermal and musculoskeletal abnormalities. First described in the Navajo population in 1991, approximately 100 cases have since been reported worldwide. While dermatologic and hematologic features are well recognized, orthopedic manifestations remain underreported. Limited literature describes bone involvement, including cases of severe osteomyelitis and chronic cellulitis. Progressive tendon fibrosis and contractures are particularly underrecognized and may lead to misdiagnosis as congenital arthrogryposis or connective tissue disorders.

### Case Description

We present two brothers, aged 20 and 16, born in India, who developed lower extremity rash, palmoplantar hyperkeratosis with nail hardening, hypotonia, and gait disturbances. Both demonstrated bilateral hamstring tightness, tight heel cords, and a stiff-legged gait consistent with progressive contractures. Genetic testing revealed a pathogenic variant in the C16ORF57 gene on chromosome 16, confirming PN. Management included intensive physiotherapy, botulinum toxin injections, and coordinated multidisciplinary care involving dermatology, orthopedics, and pediatrics.

### Discussion

This report expands the recognized phenotypic spectrum of PN by highlighting early tendon contractures as a significant manifestation. Recognition of musculoskeletal involvement alongside characteristic skin findings may facilitate earlier diagnosis and reduce misclassification. Important differential diagnoses include Rothmund-Thomson syndrome and Kindler syndrome. Despite PN being described over three decades ago, long-term survival data remain limited. Our cases demonstrate survival into early adulthood, suggesting that while PN is associated with significant morbidity related to chronic neutropenia, extended longevity is possible.

### Conclusion

PN may present with early tendon contractures indicative of a systemic fibrosing disorder. Multidisciplinary management and genetic counseling are essential for accurate diagnosis, family planning, and long-term monitoring.

